# A Giant Scapular Aneurysmal Bone Cyst in a Child

**DOI:** 10.1155/2012/327023

**Published:** 2012-05-17

**Authors:** Theodoros Beslikas, Anastasios Chytas, Andreas Christodoulou, Ioannis Gigis, Ioannis Christoforidis

**Affiliations:** 2nd Orthopaedic Department, General Hospital of Thessaloniki “G. Gennimatas”, Aristotle University of Thessaloniki, 54635 Thessaloniki, Greece

## Abstract

Aneurysmal bone cysts (ABCs) are rare benign bone tumours. Scapula is a very rare location, and the relative literature is sparse. The purpose of this study is to present a case of a giant aggressive scapular aneurysmal bone cyst in a child. A 7-year-old boy presented to our hospital with pain and a palpated mass on the right scapula. Imaging studies (radiographs computed tomography scintigraphy) were indicative of aneurysmal bone cyst. We performed curettage and bone grafting after the diagnosis was set by pathological examination through a posterior shoulder approach. Five years later, the patient has only residual signs of the lesion on radiographic control without signs of recurrence.

## 1. Introduction

Aneurysmal bone cysts (ABCs) are rare benign bone tumours that were first described by Jaffe and Lichtenstein in 1942 [1]. They account for 1% of all biopsied primary bone tumours [2] and appear as rapidly growing destructive lesions that expand the cortices [3]. ABC can exist either as a primary bone lesion (70%) or as a secondary lesion when a preexisting osseous lesion can be identified (30%) [4]. Most patients are under twenty years of age [5]. The lesions are usually present in the long bones, particularly the humerus, femur, tibia, and fibula [3, 5]. Scapula is a very rare location and accounts for 2.3% of all aneurysmal bone cyst locations [5]. The literature concerning scapular aneurysmal bone cyst is sparse. The purpose of this study is to present a case of a late presented giant scapular aneurysmal bone cyst.

## 2. Case Report

**Figure 1:**
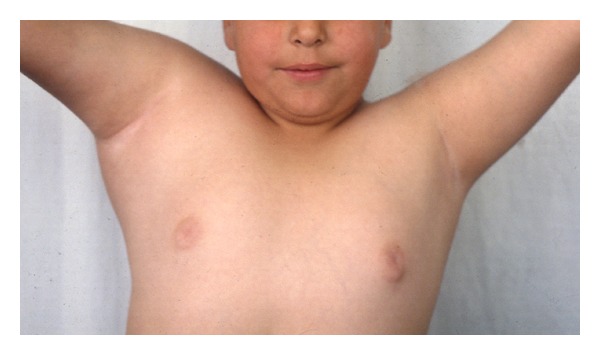
The scapula cyst raises in the axilla and limits abduction of the right shoulder.

**Figure 2:**
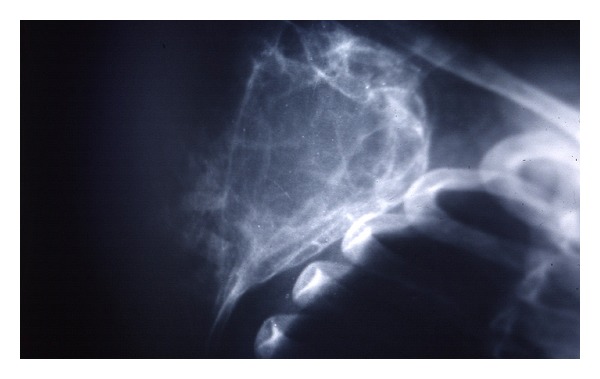
Preoperative scapular view radiograph of the right scapula.

**Figure 3:**
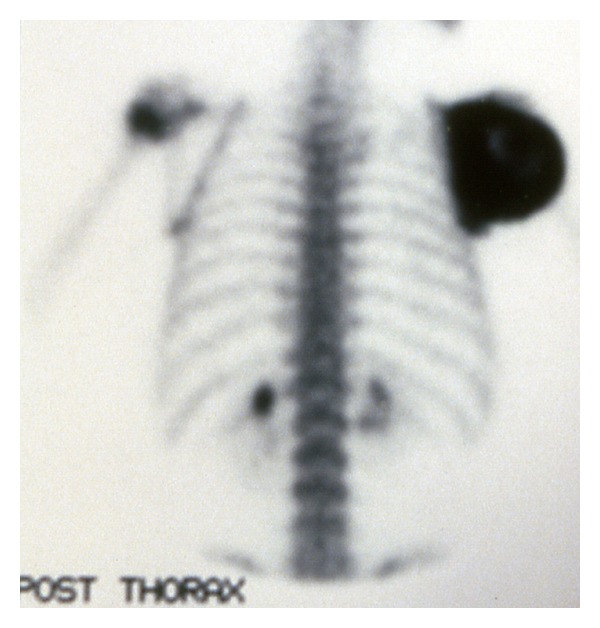
^99m^Tc-MDP bone scintigraphy. Posterior aspect.

**Figure 4:**
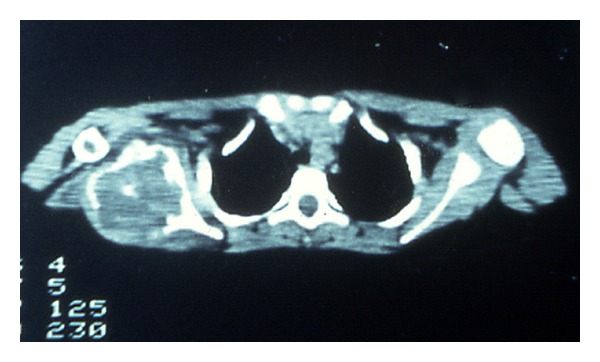
Computed tomography of the scapula.

A 7-year-old boy presented to our clinic with a palpated mass of the right scapula, discovered four months ago. The mass was enlarged fast and produced mild pain in the last three weeks. The shoulder had limited movements in comparison to the normal side and mostly abduction and forward elevation (Figure 1). The mass was hard in palpation with tenderness and extended from the posterolateral edge of the scapula. Radiographic control showed a radiolucent cystic lesion with multiple diaphragms, well-defined margins, and with expansion of the scapular cortices (Figure 2). Blood tests were normal. ^99m^Tc-MDP scintigraphy showed increased uptake of the drug mostly at the periphery of the lesion. (Figure 3) Computed tomography showed the size (oviform with 8 × 8 × 10 cm dimensions) and the extension into the axillary fold as well as the cortical defect at the posterior surface of the cyst (Figure 4). Fine-needle aspiration showed hemorrhagic fluid, but it was inconclusive.

Through a posterior shoulder approach, we took biopsy specimens for pathological examination, and we performed curettage and bone grafting. Pathological examination showed slit-like hemorrhagic spaces surrounded by fibrous septa containing spindled cells, inflammatory cells, and a lesser number of osteoclast-like multinucleated giant cells, and the diagnosis was aneurysmal bone cyst. The patient was treated with a sling for 3 weeks and with initiation of pendulum exercises from the first postoperative day. After this period, active shoulder movements were allowed without any other restriction but pain.

**Figure 5:**
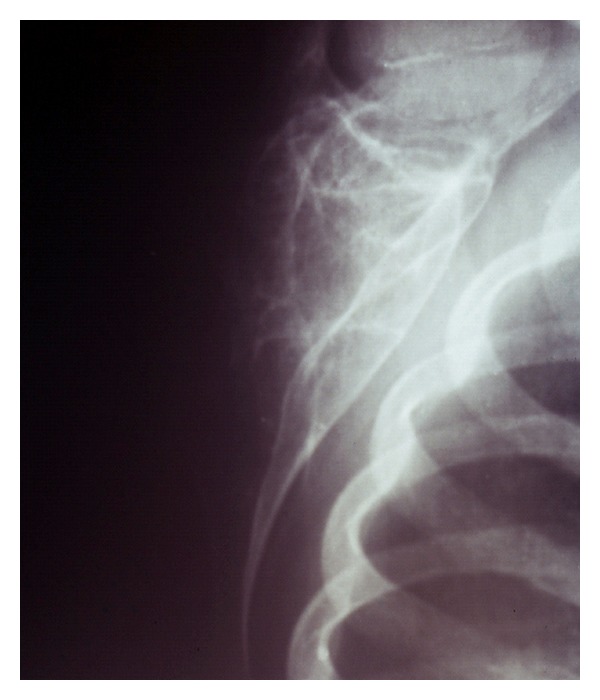
Postoperative scapular view radiograph of the right scapula. Five-year followup.

Three months postoperatively, shoulder movements were similar to those of the normal side. The patient was free of symptoms. Radiographic control showed the existence of the lesion, but it was smaller and denser. Five years postoperatively the patient was still free of symptoms, while the radiographic control showed only residual lesion signs (Figure 5).

## 3. Discussion

The pathogenesis of aneurysmal bone cysts remains controversial. They have been considered as reactive processes resulting from a local increased venous pressure and development of a dilated vascular bed within the involved bone [3, 6]. There are also many investigators who believe that at least some of the ABCs are neoplastic [3, 7, 8].

Their diagnosis is based on a thorough history, physical examination, and imaging studies [3]. In the long bones the radiographic appearance of the ABC is a subperiosteal, metaphyseal eccentric, or concentric lesion, elevating and inflating the periosteum and progressively eroding the cortex [2]. There are also many septa and ridges in the osteolytic area (honeycomb appearance) [2]. CT and MRI imaging studies are useful in evaluating soft tissue and intramedullary extension of the lesion [3], but they are not always conclusive, and ABC is sometimes added on to a list of diagnoses including eosinophilic granuloma, giant cell tumour, and unicameral bone cyst [9]. During bone scintigraphy, the increased uptake reflects the true pathologic extent of the lesion. In most cases, the abnormally increased uptake is located around the periphery of the cyst, with less activity in the center (ring-like pattern) [10].

ABCs are staged as latent that remain static or heal spontaneously, as active that grow progressively without cortical destruction and as locally aggressive that lead to cortical defects [3]. Our patient had an aggressive aneurysmal bone cyst with fast growth and posterior cortical destruction.

The treatment of aneurysmal bone cysts consists mostly of curettage and bone grafting or cementation [11]. Selective arterial embolization is an effective treatment and should be considered in lesions whose site or size makes other treatment methods difficult or hazardous [12]. En bloc resection of the cyst is the treatment of choice in eccentric lesions or in lesions arising in less essential bones like fibula or clavicle [13]. Percutaneous embolization of the cyst with an alcoholic solution of zein is another treatment option for medical teams with great experience since there are many complications [2].

Cottalorda and Bourelle [2] in a review of the literature presented the recurrence rates of the treatment of ABCs. Marginal or wide resection showed the lowest rates (4%), while curettage and bone grafting has the greatest probability for recurrence (30.6%). De Silva et al. [5] reported that 82% of recurrences are seen within 1 year of treatment, while Maurogenis et al. [14] stated that all local recurrences occur in the first two years after treatment. Thus, at least two-year followup is needed in order to exclude any local recurrence.

Scapular aneurysmal bone cysts are rare, and the literature is sparse. Kaila et al. [15] presented a literature review from 1998 to 2004 concerning shoulder girdle ABCs. Only eleven aneurysmal bone cysts were located in scapula in a total of 852 ABCs (1.3%). Scapula lesions were treated with either curettage and bone grafting or resection. They also presented 2 cases of scapula aneurysmal bone cyst in a total of 134 patients (1.5%). Megas et al. [16] presented a case of scapula aneurysmal bone cyst treated with subtotal scapula removal in a young male. Maurogenis et al. [14] presented a case of aneurysmal bone cyst located in acromion and treated with arterial embolization without recurrence. In our case, although the cyst was very extended, we performed curettage and bone grafting in order to maintain the glenohumeral joint.

As a conclusion, scapular aneurysmal bone cysts are rare. Their diagnosis remains more difficult in comparison to other locations (metaphysis of long bones), and in most instances, bone biopsy is needed. During treatment decision making, there are many options with curettage being the most common. Recurrence is a common complication after their treatment, and at least two-year followup is always necessary.
